# Risk stratification and beneficiary selection among elderly nasopharyngeal carcinoma patients from concurrent chemoradiotherapy combined with induction chemotherapy

**DOI:** 10.1002/cam4.5789

**Published:** 2023-04-16

**Authors:** Shuiqing He, Yan‐Ling Wu, Yongxiang Gao, Danjie He, Ying Huang

**Affiliations:** ^1^ Department of Radiation Oncology Sun Yat‐Sen University Cancer Center, State Key Laboratory of Oncology in South China, Collaborative Innovation Center of Cancer Medicine Guangzhou China; ^2^ Department of Radiation Oncology National Cancer Center/National Clinical Research Center for Cancer/Cancer Hospital and Shenzhen Hospital, Chinese Academy of Medical Sciences and Peking Union Medical College Shenzhen China; ^3^ Department of Data Mining and Analysis Guangzhou Tianpeng Technology Co., Ltd Guangzhou China

**Keywords:** concurrent chemotherapy, elderly, Epstein–Barr virus, induction chemotherapy, nasopharyngeal carcinoma, prognostic nomograms

## Abstract

**Objective:**

This study aims to evaluate the risk stratification among elderly Nasopharyngeal carcinoma (NPC) patients (≥60 years old) and select the beneficiaries from concurrent chemotherapy (CCRT) combined with induction chemotherapy (IC).

**Materials and Methods:**

A total of 909 elderly non‐metastatic NPC patients treated with cisplatin‐based CCRT or IC + CCRT between January 2007 and December 2016 were included. Prognostic nomograms were generated according to clinical characteristics and serum biomarkers. The survival outcomes of patients treated with CCRT versus IC + CCRT were compared in three well‐matched risk groups (high, medium, and low risk) after PSM analysis. Benefit of IC in people older or younger than 70 years and effect of different IC regimens and cycles on prognosis were analyzed.

**Results:**

Nomograms of overall survival (OS) (C‐index: 0.64, 95% CI, 0.61–0.89) and disease special survival (DSS) (C‐index: 0.65, 95% CI, 0.62–0.71) showed good prognostic accuracy. The nomogram for DSS included variables of age, gender, ACE, EBV DNA, N stage, and T stage. OS included variables of age, smoking history, ACE, ALB, EBV DNA, N stage, and T stage. The corresponding 5‐year OS rates of high, medium and low risk groups were 87.4%, 82.2%, and 60.9%, respectively (*p* < 0.001), while the 5‐year DSS rates were 92.2%, 84.3%, and 69.0%, respectively (*p* < 0.001). In the high risk group, IC + CCRT led to significantly higher 5‐year OS and DSS rate compared with CCRT (5‐year OS rate, 73.5% versus 51.8%, *p* = 0.006; 5‐year DSS rate, 81.4% versus 61.3%, *p* = 0.002). While in the medium and low risk groups, OS and DSS were not significantly different (OS: *p* = 0.259, 0.186; DSS: *p* = 0.29, 0.094). Subgroup analysis showed in the high risk group, only people younger than 70 years old could benefit from IC. TPF and IC cycles of three could lead to the best survival results.

**Conclusion:**

Compared with CCRT, OS, and DSS among high risk elderly patients were significantly improved by the addition of IC in patients younger than 70 years old. TPF and three IC cycles were recommended.

## Graphical abstract

Elderly NPC patients <70 years of age in the high‐risk group have survival benefits from CCRT, and TOF regimen, 3 cycles of induction chemotherapy, has significantly improved survival.

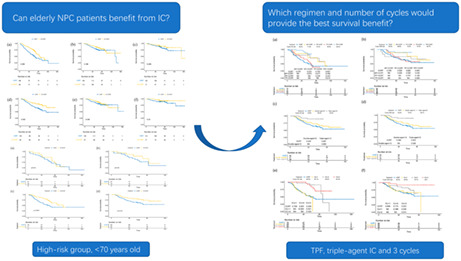

## 1 INTRODUCTION

Nasopharyngeal carcinoma (NPC) is a sort of squamous original head and neck cancer with an unbalanced endemic distribution, especially prevalent in Southern China.^1^ The peak age of NPC incidence is from 40 to 60 years old and subsequently reduced.^2^ Due to the lack of obvious early symptoms, 70% of patients are in advanced stage (Stages III‐IV) at the time of diagnosis.^3^, ^4^ The NCCN guideline recommends the use of concurrent chemotherapy (CCRT) combined with induction chemotherapy (IC) for the treatment of locally advanced NPC (T1, N1‐3; T2‐4, and N) patients. Previous studies have shown that CCRT may achieve local tumor control due to its synergistic or additive effect with RT,^5^ while IC may gain survival benefit by eradicating micro‐metastasis.^6^

Several randomized controlled trials have demonstrated that IC added to CCRT is the standard‐of‐care curative method for locoregionally advanced NPC patients; however, with a limited sample size of elderly patients: 18–59 years old^7^, ^8^; 19–66 years old, with a median age of 44 in IC + CCRT group and 42 in CCRT group^9^; 31–70 years old, with a median age of 45 in the control arm and 50 in neoadjuvant arm^10^; the median age of 46 in IC + CCRT group and 48 in CCRT group.^11^ Elderly patients are susceptible to being concomitant with declined functional status and increased comorbidities.^12^, ^13^, ^14^ The tolerance to CCRT combined with IC is vital for elderly patients associated with reduced organ function, less social support, comorbidity, and poorer performance status.^14^ Baseline health and function should be considered when deciding whether to undergo chemotherapy. Comparative benefits and risks should be also discussed carefully.^15^ Wang reported that compared with CCRT alone, there was no improvement in 5‐year DSS, OS, DFFS, and LRFFS rates for patients receiving IC + CCRT, nor with a higher cumulative incidence of grade 3–4 toxicity among elderly patients with advanced NPC.^16^ However, this study had a small sample size and lacked essential clinical risk factors, such as EBV DNA. In addition, no more research is available to confirm the results of this study. Therefore, the treatment efficacy of IC in elderly NPC patients is not promising.

To select the beneficiaries from CCRT combined with IC for elderly NPC patients, a real‐world, large‐scale study was initiated with a nomogram model based on clinical risk factors to divide patients into high, medium, and low risk groups. For each well‐matched risk group after propensity score matching (PSM), survival outcomes of CCRT, and IC + CCRT were compared to evaluate the benefit of IC in elderly patients.

## 2 MATERIALS AND METHODS

### 2.1 Study population

This study used an NPC‐specific database derived from the well‐established big data platform of Sun Yat‐sen University Cancer Center, SYSUCC, (Yi‐du Cloud Technology Ltd., Beijing, China), whose detailed introduction has been provided in a previous study.^17^ A specific subset of endemic cases was represented by this large real‐world dataset, which is located in Southern China.

Eligible patients were enrolled according to the following criteria: initially diagnosed non‐distant metastasis disease; age ≥60 years old; treated with CCRT with or without IC; detailed medical history; complete basic laboratory experiments, such as plasma EBV DNA, lactate dehydrogenase (LDH), hemoglobin (HGB), and C‐reactive protein (CRP). A total of 909 patients meeting the criteria diagnosed between April 2007 and December 2016 were included. This retrospective research was approved by Institutional Review Board (IRB‐approved number, SL‐B2021‐474‐02) of SYSUCC, GuangZhou, China. Clinical information was anonymous, and the requirement of informed consent was waived. The raw data have been uploaded to the public platform of Research Data Deposit (http://www.researchdata.org.cn) and the RDD‐approved number is RDDA2022874324.

### 2.2 Diagnosis and treatment

Pre‐treatment evaluation was undergone by all patients, including chest and abdominal computed tomography (CT), nasopharynx and neck magnetic resonance imaging (MRI), fiberoptic nasopharyngoscopy, hematologic and biochemical test, complete case record, and physical examination. 18F‐fluorodeoxyglucose positron emission tomography (PET) and CT (PET‐CT), or whole‐body bone scan (ECT) was adopted to determine the TNM stage. The real‐time quantitative polymerase chain‐reaction (PCR) assay was used to test pre‐treatment plasma EBV DNA titer for each patient. All patients were restaged according to the eighth edition of the American Joint Committee on Cancer /Union for International Cancer Control (AJCC/UICC) manual. Stratified multi‐therapeutic principles established by SYSUCC (see Supplementary Materials) were employed for all NPC patients. IC regimens included TP (cisplatin 80 mg/m^2^, docetaxel 80 mg/m^2^), PF (cisplatin 80 mg/m^2^, with 5‐fluorouracil 800 mg/m^2^/day over 5 days), TPF (docetaxel 60 mg/m^2^, cisplatin 60 mg/m^2^, 5‐fluorouracil 600 mg/m^2^ over 5 days), GP (cisplatin 80 mg/m^2^ on day 1, gemcitabine 1000 mg mg/m^2^ on day 1 and 8), administrated every 3 weeks. During radiation therapy, concurrent cisplatin was given weekly (40 mg/m^2^) or in weeks 1, 4, and 7 (80 or 100 mg/m^2^).

### 2.3 Evaluation of comorbidity status

Comorbidity has been reported to be an important factor in selecting treatment choices and affecting survival outcomes for cancer patients.^18^, ^19^ Assessment 27 (ACE‐27), including 27 different comorbidities from different organs and systems of the body as a scoring tool specifically for cancer patients, was used as a uniform standard for assessing comorbidity status and determining health severity.^20^ This was calculated by reviewing patients' medical records and was classified into severe (score 3), moderate (score 2), mild (score 1), or none (score 0), based on ACE 27. The ACE score is defined as severe when two or more moderate diseases occur in different disease groups, organs, or systems. The comorbidity that lacks specific information was designated as mild according to the Comorbidity Coding Book. These scores were used to determine the overall comorbidity status.

### 2.4 Follow‐up and end point

Patients were reviewed every 3 months during the first 2 years and every 6 months during the next 3 years, and then every year thereafter. Plasma EBV DNA, fiberoptic nasopharyngoscopy, and complete clinical examination were performed routinely during the visit. MRI, abdominal ultrasound, contrast imaging, cytological biopsy, PET‐CT, or ECT were recommended for patients with clinically suspected metastases. The primary end point was disease special survival (DSS), defined as the time from initial treatment until the date of cancer‐related mortality; the second end point was overall survival (OS), defined as the time from initial treatment until the date of death or the last follow‐up.

### 2.5 Statistical analysis

Continuous variables were converted into categorical variables based on clinical cut‐off points (CRP, ALB) and according to prior studies,^21^, ^22^ confirming that the optimal cut‐off value of EBV DNA was 2000 copies/mL. Integrating pre‐treatment plasma EBV DNA into the TNM staging system provided better hazard discrimination and outcome prediction than the 8th TNM staging system alone, which may be able to guide individualized treatment strategies in NPC. LDH and HGB were classified as normal and abnormal ranges based on clinical cut‐off points. OS and DSS rates were calculated using the Kaplan–Meier method and compared using the stratified log‐rank test. A univariate Cox proportional hazard model was used to analyze potential factors, and the multivariable Cox regression analysis included variables with *p* < 0.05. Adjusted hazard ratios (HRs) and 95% confidence intervals (CIs) of the validated predictors for DSS and OS were presented using forest plots. Statistically significant variables after multivariable Cox regression analysis were then used to generate nomograms. A backward step‐down selection process with Akaike information criteria was used for the selection of the final prediction model.^23^ The performance of nomograms was evaluated by concordance index (C‐index), and bootstraps with 1000 resamples were applied.

All patients were divided into three groups (high, medium, and low risk) according to tertiles of the total risk scores generated by nomograms for OS and DSS. And the survival outcomes of patients who received CCRT alone or IC + CCRT were compared in each risk group to select IC beneficiaries. Propensity score matching (PSM) with a stringent caliper of 0.2 was performed to reduce the influence of potential confounding factors on selection bias. Categorical variables in different groups were compared by the Fisher's exact test or Chi‐square test. All analyses were two‐sided with a 95% level of significance. All statistical models were generated using R software, version 4.1.2 (http://www.r‐project.org/).

## 3 RESULTS

### 3.1 Baseline characteristics of patients

A total of 909 eligible patients between January 2007 and December 2016 were included in this study. Table 1 shows the baseline characteristics of all patients. The median age was 63 years old (range: 60–79), and the sex ratio (M/F) was 3.21:1 (693 males and 216 females). Nonkeratinizing undifferentiated NPC (WHO type III) accounted for the majority (97.7%) of all pathologic types. In total, 463 (50.9%) patients had pre‐treatment plasma EBV DNA >2000 copies/mL. The proportions of T stage, N stage, and overall clinical stage in all patients were T1 (7.5%), T2 (12.2%), T3 (50.9%), T4 (29.4%); N0 (11.4%), N1 (48.1%), N2 (26.8%), N3 (13.6%); stage II (12.1%), III (48.1%), IV (39.8%). In this study, there were 46 patients with an ACE‐27 score of 2, 400 with a score of 1, and 463 with a score of 0. Of all patients, 387 (42.6%) patients received IC + CCRT, and 522 (57.4%) patients received CCRT alone. Specific information on patients receiving treatment is provided in Supporting Information. The median follow‐up time was 61.8 months (1.6–170.1), 267 (29.4) died during the study period, and 187 patients (20.6%) died because of tumor. The overall 3‐year and 5‐year OS were 86.4% and 77.0%, while the 3‐year and 5‐year DSS were 88.7% and 82.0%.

**TABLE 1:** Baseline characteristics and univariate analysis of OS and DSS in the 909 elderly NPC patients.

Characteristics	No. (%)^a^	Univariate analysis			
		OS		DSS	
Gender		HR (95% CI)^b^	*p*‐value	HR (95% CI)^b^	*p*‐value
Male	693 (76.2%)				
Female	216 (23.8%)	0.68 (0.50, 0.93)	0.012	0.67 (0.46, 0.97)	0.029
Age					
60–69	821 (90.3%)				
>=70	88 (9.7%)	2.32 (1.65, 3.25)	<0.001	2.19 (1.46, 3.29)	<0.001
WHO					
3	888 (97.7%)				
1 & 2	21 (2.3%)	1.33 (0.66, 2.69)	0.4	1.73 (0.81, 3.68)	0.2
Smoking					
No	519 (57.1%)				
Yes	390 (42.9%)	1.34 (1.05, 1.70)	0.018	1.23 (0.93, 1.64)	0.2
Drinking					
No	744 (81.8%)				
Yes	165 (18.2%)	1.09 (0.81, 1.48)	0.6	0.87 (0.59, 1.29)	0.5
ACE‐27					
0	463 (50.9%)				
1	400 (44.0%)	0.92 (0.72, 1.19)		0.85 (0.63, 1.16)	
2	46 (5.1%)	2.25 (1.40, 3.61)	0.005	2.28 (1.34, 3.88)	0.006
Family history					
No	691 (76.0%)				
Yes	218 (24.0%)	0.77 (0.58, 1.04)	0.078	0.76 (0.54, 1.09)	0.12
T stage					
T1‐2	179 (19.7%)				
T3	463 (50.9%)	1.33 (0.92, 1.91)		1.18 (0.77, 1.81)	
T4	267 (29.4%)	2.03 (1.40, 2.95)	<0.001	2.04 (1.32, 3.15)	<0.001
N stage					
N0‐1	541 (59.5%)				
N2	244 (26.8%)	1.17 (0.89, 1.55)		1.35 (0.97, 1.88)	
N3	124 (13.6%)	1.66 (1.19, 2.31)	0.015	1.79 (1.21, 2.64)	0.012
Stage					
II	110 (12.1%)				
III	437 (48.1%)	1.12 (0.72, 1.75)		1.15 (0.67, 1.98)	
IV	362 (39.8%)	1.92 (1.24, 2.97)	<0.001	2.1 (1.24, 3.56)	<0.001
EBV‐DNA (copy/mL)					
<=2000	446 (49.1%)				
>2000	463 (50.9%)	1.76 (1.37, 2.26)	<0.001	2.37 (1.73, 3.24)	<0.001
HGB (g/L)^b^					
Abnormal	61 (6.7%)				
Normal	848 (93.3%)	1.14 (0.74, 1.75)	0.6	1.03 (0.59, 1.78)	>0.9
ALB(g/L)^b^					
35–55	778 (85.6%)				
<35	131 (14.4%)	1.65 (1.22, 2.23)	0.002	1.42 (0.97, 2.07)	0.081
LDH^b^					
Abnormal	71 (7.8%)				
Normal	838 (92.2%)	1.26 (0.83, 1.90)	0.3	1.22 (0.74, 2.01)	0.5
CRP (g/L)^b^					
0–8.2	655 (72.1%)				
>8.2	254 (27.9%)	1.45 (1.12, 1.88)	0.006	1.45 (1.07, 1.97)	0.019
IC regimen^c^					
TPF	148 (38.2%)				
TP	128 (31.1%)	1.13 (0.70, 1.80)		0.93 (0.54, 1.60)	
PF	86 (22.2%)	1.70 (1.07, 2.70)		1.46 (0.86, 2.50)	
GP	25 (6.5%)	0.71 (0.22, 2.31)	0.106	0.53 (0.13, 2.24)	0.272
IC cycles^c^					
4	19 (4.4%)				
3	144 (37.2%)	0.63 (0.30, 1.35)		0.53 (0.23, 1.22)	
2	183 (47.3%)	0.60 (0.28, 1.27)		0.46 (0.20, 1.05)	
1	41 (10.6%)	1.28 (0.56, 2.94)	0.049	1.20 (0.49, 2.92)	0.02

Abbreviations: ACE, adult comorbidity evaluation; ALB, albumin; CI, confidence interval; CRP, C‐reactive protein; DSS, disease‐special survival; EBV, Epstein–Barr virus; HR hazard ratio; HR, hazard ratio; LDH, lactate dehydrogenase; NPC, nasopharyngeal carcinoma; OS, overall survival; WHO, World Health Organization.

^a^ Percentages may not add up to 100% due to rounding.

^b^ All variables were measured before treatment.

^c^ These variables were measured only in patients who received induction chemotherapy.

### 3.2 Development of nomograms for OS and DSS

**FIGURE 1:**
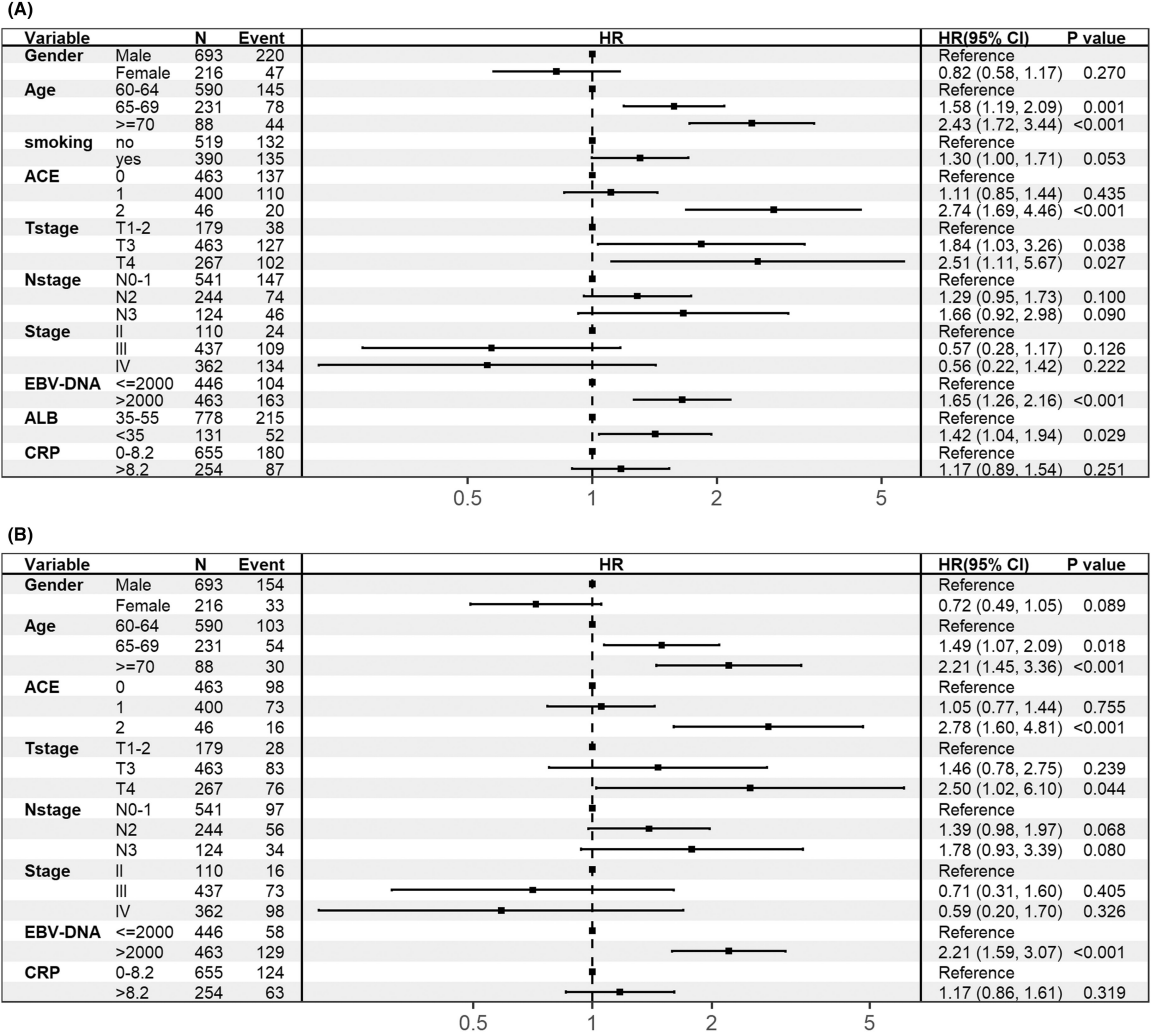
Forest plots describing the multivariate association of clinical and biochemical indicators with OS (A) and DSS (B). ACE, adult comorbidity evaluation; ALB, albumin; CRP, C‐reactive protein; DSS, disease‐special survival; EBV, Epstein–Barr virus; OS, overall survival.

**FIGURE 2:**
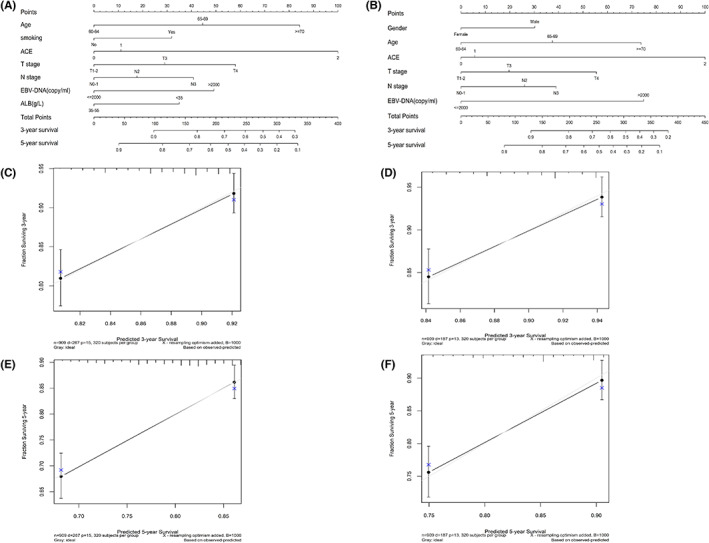
Prognostic nomograms (A, B) and calibration plots of 3‐year (C, D) and 5‐year (E, F) in elderly NPC patients. The left panel shows the nomogram and calibration plots for OS (A, C, E); the right panel shows the nomogram and calibration plots for DSS (B, D, F). ALB, albumin; DSS, disease‐special survival; EBV, Epstein–Barr virus; OS, overall survival.

Figure 1 shows the results of multivariate analysis. As a result, gender, age, smoking history, ACE score, EBV‐DNA, ALB, CRP, T stage, N stage, and overall clinical stage were proven to be significant indicators for OS (Figure 1A); gender, age, ACE score, EBV‐DNA, CRP, T‐stage, and N‐stage were proven to be significant indicators for DSS (Figure 1B). Nomograms and corresponding calibration plots for OS and DSS are shown in Figure 2. All the above‐mentioned variables were used to generate prognostic nomograms for OS (Figure 2A) and DSS (Figure 2B), with corresponding C‐index values of 0.64 (95% CI, 0.61–0.89) and 0.65 (95% CI, 0.62–0.71). Additionally, calibration plots for probabilities of 3‐year and 5‐year OS (Figure 2C,E) and DSS (Figure 2D,F) showed good consistency with nomogram predictions for OS.

### 3.3 Risk stratification for OS and DSS

**FIGURE 3:**
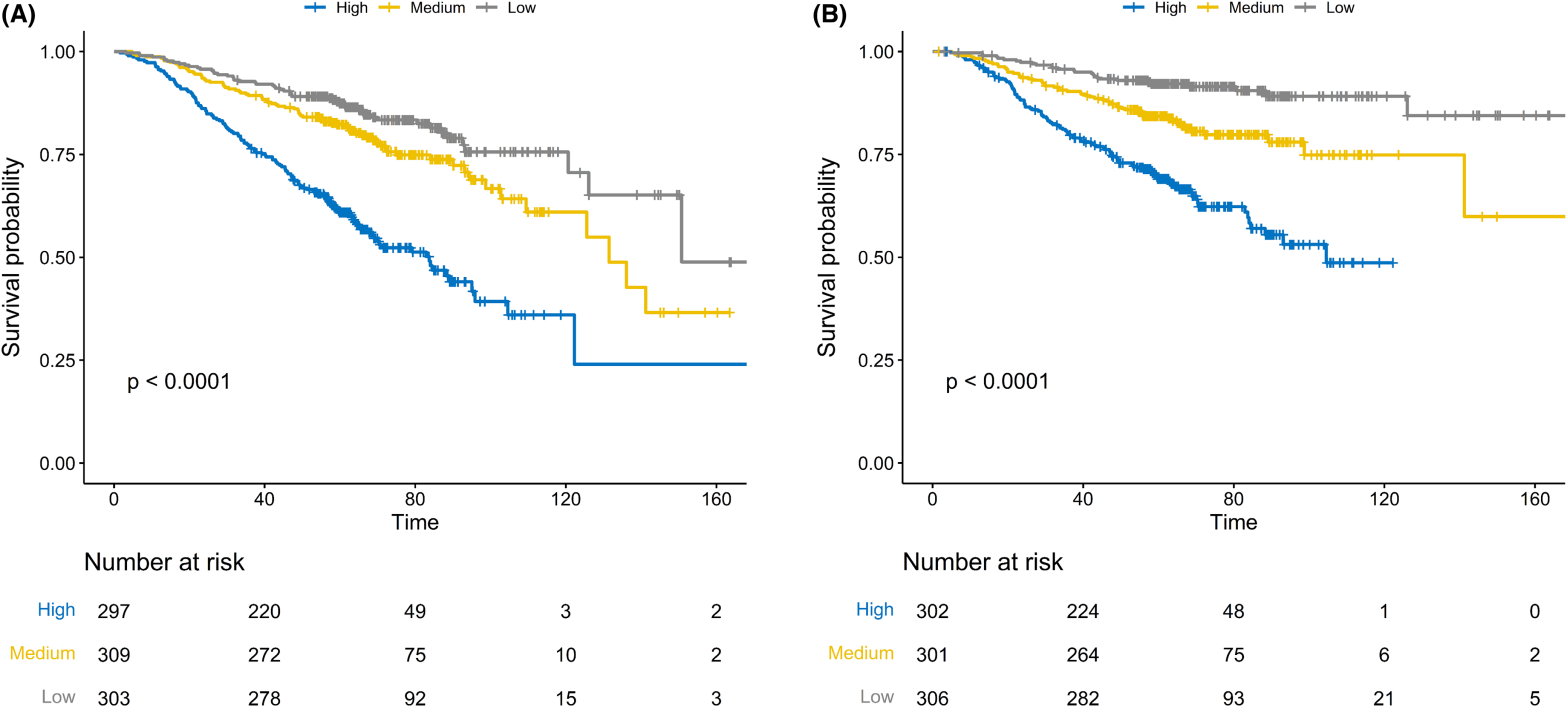
Kaplan–Meier survival curves for OS (A) and DSS (B) in elderly NPC patients, stratified by tertiles of total risk scores generated from nomograms.

Tertiles of total risk scores were utilized to divide patients into high (302 patients), medium (301 patients), and low risk (306 patients) groups. Baseline clinical characteristics of three risk groups for DSS before PSM analysis are shown in Table 2. Table S1 shows the basic information about three risk groups for OS. Survival curves presented significant discrimination among the three risk groups, as is shown in Figure 3. The corresponding 5‐year OS rate of low, medium, and high risk group was 87.4%, 82.2%, and 60.9%, respectively (*p* < 0.001, Figure 3A), 5‐year DSS rate was 92.2%, 84.3%, and 69.0%, respectively (*p* < 0.001, Figure 3B).

**TABLE 2:** The baseline characteristics of high, intermediate, and low‐risk groups for DSS in patients treated with IC plus CCRT or CCRT alone before propensity score matching.

Characteristics	High‐risk group	Intermediate‐risk group	Low‐risk group	*p*‐value
	*n* = 302	*n* = 301	*n* = 306	
	No. %^a^	No. %^a^	N0. %^a^	
Gender				<0.001
Male	264 (87.4%)	216 (71.8%)	213 (69.6%)	
Female	38 (12.6%)	85 (28.2%)	93 (30.4%)	
Age				<0.001
60–64	137 (45.4%)	209 (69.4%)	244 (79.7%)	
65–69	109 (36.1%)	63 (20.9%)	59 (19.3%)	
>=70	56 (18.5%)	29 (9.6%)	3 (1.0%)	
WHO				0.559
3	293 (97.0%)	296 (98.3%)	299 (97.7%)	
1&2	9 (3.0%)	5 (1.7%)	7 (2.3%)	
Smoking				0.259
No	161 (53.3%)	179 (59.5%)	179 (58.5%)	
Yes	141 (46.7%)	122 (40.5%)	127 (41.5%)	
Drinking				0.443
No	242 (80.1%)	253 (84.1%)	249 (81.4%)	
Yes	60 (19.9)	48 (15.9%)	57 (18.6%)	
ACE				<0.001
0	153 (50.7%)	171 (56.8%)	139 (45.4%)	
1	116 (38.4%)	117 (38.9%)	167 (54.6%)	
2	33 (10.9%)	13 (4.3%)	0	
Family history				0.169
No	241 (79.8%)	223 (74.1%)	227 (74.2%)	
Yes	61 (20.2%)	78 (25.9%)	79 (25.8%)	
T stage				<0.001
T1‐2	22 (7.3%)	75 (24.9%)	82 (26.8%)	
T3	111 (36.8%)	169 (56.1%)	183 (59.8%)	
T4	169 (56.0%)	57 (18.9%)	41 (13.4%)	
N stage				<0.001
N0‐1	130 (43.0%)	169 (56.1%)	242 (79.1%)	
N2	99 (32.8%)	90 (29.9%)	55 (18.0%)	
N3	73 (24.2%)	42 (14.0%)	9 (2.9%)	
Stage				<0.001
II	6 (2.0%)	41 (13.6%)	63 (20.6%)	
III	74 (24.5%)	169 (56.1%)	194 (63.4%)	
IV	222 (73.5%)	91 (30.2%)	49 (16.0%)	
EBV‐DNA (copy/mL) ^b^				<0.001
<=2000	31 (10.3%)	112 (37.2%)	303 (99.0%)	
>2000	271 (89.7%)	189 (62.8%)	3 (1.0%)	
HGB (g/L)^b^				0.787
Abnormal	283 (93.7%)	282 (93.7%)	283 (92.5%)	
Normal	19 (6.3%)	19 (6.3%)	23 (7.5%)	
ALB(g/L)^b^				0.014
35–55	244 (80.8%)	263 (87.4%)	271 (88.6%)	
<35	58 (19.2%)	38 (12.6%)	35 (11.4%)	
LDH^b^				0.003
Abnormal	31 (10.3%)	29 (9.6%)	11 (3.6%)	
Normal	271 (89.7%)	272 (90.4%)	295 (96.4%)	
CRP(g/L)^b^				<0.001
0‐8.2	186 (61.6%)	222 (73.8%)	247 (80.7%)	
>8.2	116 (38.4%)	79 (26.2%)	59 (19.3%)	
Treatment				<0.001
IC plus CCRT	172 (57.0%)	130 (43.2%)	85 (27.8%)	
CCRT	130 (43.0%)	171 (56.8%)	221 (72.2%)	

Abbreviations: ACE, adult comorbidity evaluation; ALB, albumin; CRP, C‐reactive protein; DSS, disease‐special survival; EBV, Epstein–Barr virus; HR, hazard ratio; LDH, lactate dehydrogenase; NPC, nasopharyngeal carcinoma; OS, overall survival; WHO, World Health Organization.

^a^ Percentages may not add up to 100% due to rounding.

^b^ All variables were measured before treatment.

### 3.4 Survival benefits of CCRT combined with IC in each risk group

Validated variables obtained after multivariate analysis of OS (age, smoking history, T stage, N stage, EBV‐DNA, ACE, ALB) and DSS (gender, age, T stage, N stage, EBV‐DNA, ACE) were selected for PSM analysis. The baseline patient characteristics before PSM analysis were unbalanced (Table 2). In the high risk group for OS, there were more patients staged with T3‐4, N3, more patients with elevated EBV‐DNA and lower ALB, more patients with an ACE score of 2, and more patients receiving IC, compared with the medium and low risk groups (all *p* < 0.001). This imbalance also existed in three risk groups for DSS. Three well‐matched risk groups after PSM analysis were generated to perform a survival comparison of patients receiving CCRT alone versus IC + CCRT in each group (all *p* > 0.05). Baseline characteristics are listed in Table 3. See Table S2 for information in OS.

**TABLE 3:** The baseline characteristics of the patients treated with IC plus CCRT or CCRT alone in each risk group for DSS after PSM analysis.

Characteristic	High‐risk group			Intermediate‐risk group			Low‐risk group		
	CCRT *N* = 116	IC + CCRT *N* = 95	*p*‐value^a^	CCRT *N* = 130	IC + CCRT *N* = 97	*p*‐value^a^	CCRT *N* = 113	IC + CCRT *N* = 77	*p*‐value^a^
Gender			0.2			0.6			0.3
Male	97 (84%)	85 (89%)		92 (71%)	72 (74%)		88 (78%)	55 (71%)	
Female	19 (16%)	10 (11%)		38 (29%)	25 (26%)		25 (22%)	22 (29%)	
Age			0.2			0.5			0.4
60–64	39 (34%)	43 (45%)		92 (71%)	75 (77%)		97 (86%)	70 (91%)	
65–69	50 (43%)	37 (39%)		33 (25%)	20 (21%)		16 (14%)	7 (9.1%)	
>=70	27 (23%)	15 (16%)		5 (3.8%)	2 (2.1%)		0 (0%)	0 (0%)	
ACE			0.8			0.9			0.2
0	55 (47%)	49 (52%)		74 (57%)	57 (59%)		54 (48%)	30 (39%)	
1	49 (42%)	36 (38%)		49 (38%)	36 (37%)		59 (52%)	47 (61%)	
2	12 (10%)	10 (11%)		7 (5.4%)	4 (4.1%)		0 (0%)	0 (0%)	
T stage			0.4			0.9			0.4
T1‐2	9 (7.8%)	4 (4.2%)		36 (28%)	25 (26%)		25 (22%)	18 (23%)	
T3	51 (44%)	38 (40%)		67 (52%)	53 (55%)		72 (64%)	43 (56%)	
T4	56 (48%)	53 (56%)		27 (21%)	19 (20%)		16 (14%)	16 (21%)	
N stage			>0.9			0.4			0.4
N0‐1	49 (42%)	42 (44%)		77 (59%)	50 (52%)		86 (76%)	56 (73%)	
N2	37 (32%)	30 (32%)		39 (30%)	32 (33%)		24 (21%)	16 (21%)	
N3	30 (26%)	23 (24%)		14 (11%)	15 (15%)		3 (2.7%)	5 (6.5%)	
EBV‐DNA (copy/mL)			0.9			0.3			>0.9
<=2000	13 (11%)	10 (11%)		44 (34%)	27 (28%)		113 (100%)	77 (100%)	
>2000	103 (89%)	85 (89%)		86 (66%)	70 (72%)		0 (0%)	0 (0%)	

^a^ Pearson's Chi‐squared test; Fisher's exact test.

Abbreviations: ACE, adult comorbidity evaluation; EBV, Epstein–Barr virus.

**FIGURE 4:**
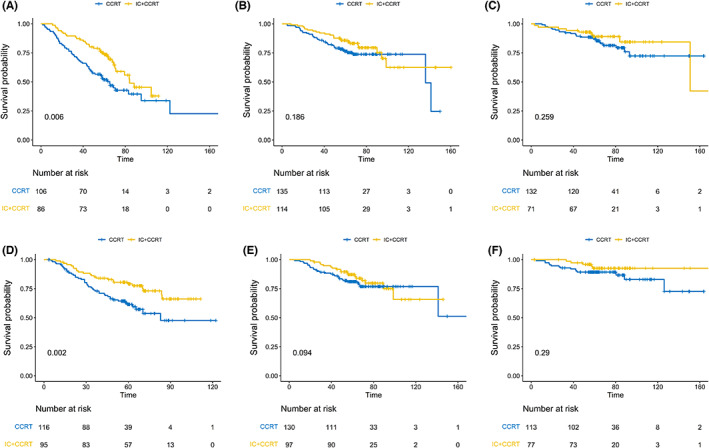
Kaplan–Meier curves of OS and DSS in three risk groups with IC plus CCRT or CCRT alone. OS: high‐risk group (A), intermediate‐risk group (B), and low‐risk group (C); DSS: high‐risk group (D), intermediate‐risk group (E), and low‐risk group (F).

Figure 4 showed the survival benefits of CCRT combined with IC in each risk group. For patients receiving IC + CCRT, there was a significant improvement for OS and DSS in the high risk group, compared to CCRT alone (5‐year OS rate, 73.5% versus 51.8%, Figure 4A, *p* = 0.006; 5‐year DSS rate, 81.4% versus 61.3%, *p* = 0.002, Figure 4D). But IC + CCRT seemed not to improve OS and DSS in the medium (Figure 4B,E) and low risk groups (Figure 4C,F). The 5‐year OS rate for patients receiving CCRT alone and IC + CCRT was 89.1% and 87.7% in the low risk group (*p* = 0.259); 85.9% and 76.4% in the medium risk group (*p* = 0.186). The 5‐year DSS rate for patients receiving CCRT alone and IC + CCRT was 92.7% and 89.3% in the low risk group (*p* = 0.29); 87.2% and 81.1% in the medium risk group (*p* = 0.094). Figure S1 showed the 5‐year DMFS and LRRFS rate in the three risk groups after matching and the corresponding survival curves.

### 3.5 Benefit of IC in people older or younger than 70 years

**FIGURE 5:**
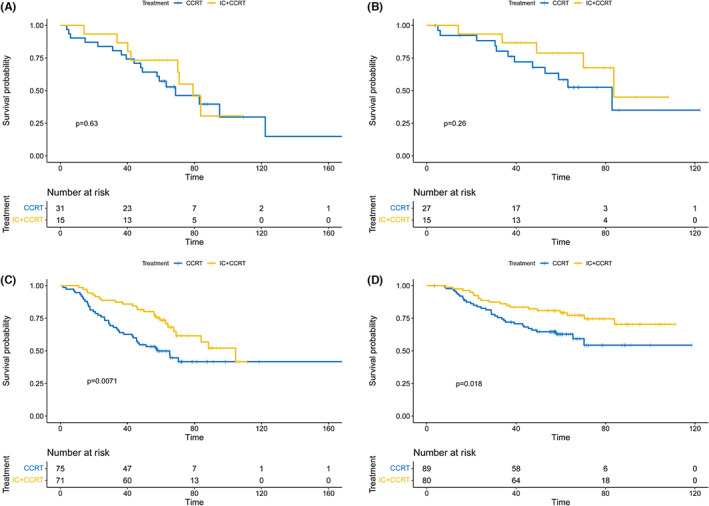
Kaplan–Meier curves of OS and DSS in patients above or younger than 70 years in the high‐risk group with IC plus CCRT or CCRT alone. OS: over 70 years (A), under 70 (C); DSS: over 70 years (B), under 70 (D).

To carefully select the beneficiaries of induction chemotherapy, we compared the difference in survival between CCRT versus IC in the high risk groups of ≥70 and <70 years of age for OS and DSS after PSM analysis. In patients over 70 years of age, the 5‐year OS rate of patients receiving IC or CCRT alone was 73.3% versus 57.3%, *p* = 0.63, the 5‐year DSS rate was 78.8% versus 58.4%, *p* = 0.26, the survival curve was shown in Figure 5A (OS) and Figure 5B (DSS). In patients younger than 70 years old, the 5‐year OS rate of patients receiving IC or CCRT alone was 79.2% versus 62.8%, *p* = 0.007, the 5‐year DSS rate was 73.5% versus 51.8%, *p* = 0.018, the survival curve was shown in Figure 5C (OS) and Figure 5D (DSS).

### 3.6 Effect of different IC regimens and cycles on prognosis

**FIGURE 6:**
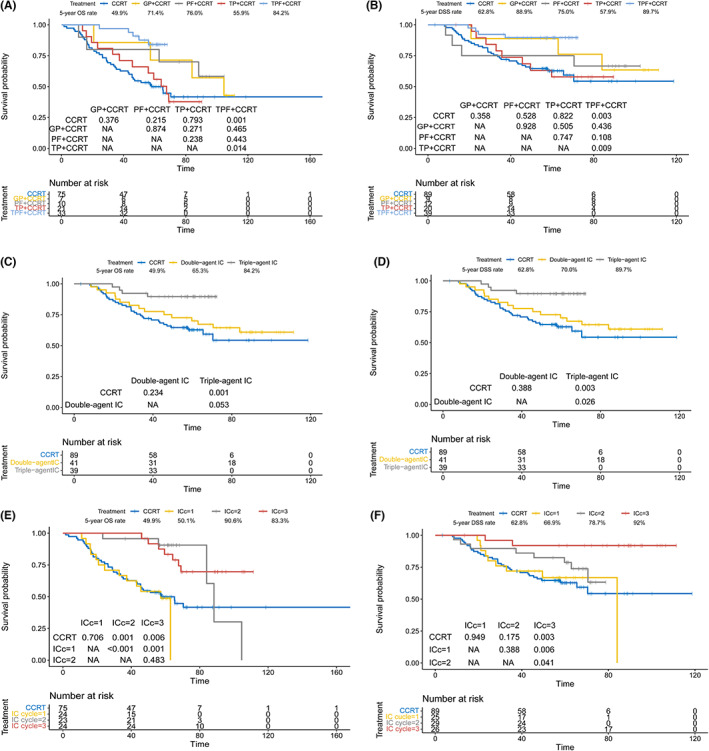
Kaplan–Meier curves of OS and DSS and the corresponding 5‐year survival rate for different IC regimens and cycles in the high‐risk group of people under 70 with IC plus CCRT or CCRT alone. OS: different IC regimens (A), double or triple‐agent IC (C), different IC cycles (E); DSS: different IC regimens (B), double or triple‐agent IC (D), different IC cycles (F).

As we summarized in the Supporting information, patients in our study received IC regimens of TPF, PF, TP, GP, and received IC cycles (ICc) of 1, 2, 3, or 4. Univariate cox analysis of IC regimens and cycles on OS and DSS was performed, the result was listed in Table 1. Considering that only patients younger than 70 years of age in the high risk group benefited from induction chemotherapy. We compared the survival of each regimen and each cycle in patients younger than 70 years of age in the matched OS and DSS high risk groups. The survival curve and 5‐year survival rate of patients receiving GP, TP, PF, and GP was summarized in Figure 6A (OS) and Figure 6B (DSS). The survival curve and 5‐year survival rate of patients receiving double‐agent IC (TP, PF, and GP) or triple‐agent IC (TPF) was summarized in Figure 6C (OS) and Figure 6D (DSS). The survival curve and 5‐year survival rate of patients receiving ICc of 1, 2, and 3 (no patients receiving 4 cycles IC after PSM analysis in these two groups) was summarized in Figure 6E (OS) and Figure 6F (DSS). Comparison of each regimen and each cycle and the corresponding *p*‐value was listed in Figure 6 as well.

## 4 DISCUSSION

IC has not been well defined in elderly NPC patients. This study is the first attempt to evaluate risk stratification and select beneficiaries in a large cohort. Nomograms are based on clinical and biochemical indicators and patients were divided into three risk groups. The high‐risk group has better survival benefits of IC + CCRT compared with CCRT alone. Thus, the high risk group in nomograms may be the IC beneficiaries in elderly NPC patients.

Sun conducted a phase III randomized controlled trial and concluded the 5‐year OS of 85.6% in the TPF (cisplatin, fluorouracil, and docetaxel) IC + CCRT group, while 77.7% in the CCRT group after a median follow‐up of 71.5 months.^7^, ^8^ Yang's phase III, multicenter randomized controlled trial reported the 5‐year OS was 80.8% in the PF IC + CCRT group; 76.8% in the CCRT alone group.^9^ In this study, the overall 5‐year OS in CCRT alone and IC + CCRT groups was 75.0% and 79.0%, respectively, which was worse than clinical trials that included participants younger than 60 years old.^7^, ^8^ This is consistent with the previous findings that age is associated with prognosis. An older age at diagnosis and worse outcomes were related to a higher risk of NPC‐associated mortality, according to the population‐based analysis.^24^, ^25^ When patients received chemotherapy, the benefits may be offset by substantial adverse effects on their normal function.^26^, ^27^, ^28^ The overall 5‐year DSS in CCRT alone and IC + CCRT groups was 81.3% and 82.9%, respectively. However, Wang's retrospective study targeted at elderly NPC patients showed that the 5‐year OS rate in IC + CCRT and CCRT alone groups was 71.8% and 60.5%, respectively. The 5‐year DSS rate in IC + CCRT and CCRT alone groups was 75.3% and 66.7%, respectively.^16^ The survival difference may be related to IC regimens: TP in Wang's study, while a variety of TP, PF, and TPF in this study. Our study showed that TPF was associated with significantly better survival than TP in the high risk group, but with a small sample size. Liu^29^ reported that for 1879 NPC patients receiving induction chemotherapy, TPF was related to a significant increase of the 5‐year OS (88.1% vs. 80.7%; *p* = 0.042) and DSS (88.5% vs. 80.7%; *p* = 0.021) compared with the PF group. The heterogeneity of IC regimen and dosage exits due to the retrospective study design. Another reason might be that the older patients included in Wang's study were stage III and IVb, whereas our study included stage II patients as well. Our study concluded that clinical stage was a significant indicator for OS, earlier stage might lead to a better prognosis. Wang reported that IC + CCRT had a high cumulative incidence of grade 3–4 toxicities while no improvements in 5‐year OS and DSS rate compared with CCRT alone and CCRT alone was the standard of treatment in elderly NPC patients. Through risk stratification, patients in the high risk group in our study who received IC had a significant better prognosis than those who received CCRT, except for those above 70 years old. Thus, we still recommended IC in the elderly NPC patients <70 years old in the high risk group.

Given the impact of mortality to which elder people are vulnerable such as hypertension, heart disease and diabetes, DSS was set as the primary endpoint. Univariate analysis showed that gender, age, ACE, T stage, N stage, clinical stage, EBV DNA, and CRP were prognostic indicators for elder NPC patients, suggesting that early detection and diagnosis may provide the best chance of survival. The nomogram for DSS included variables of gender, age, ACE, EBV DNA, T stage, and N stage. The nomogram for OS included variables of age, smoking history, ACE, EBV DNA, ALB, T stage, and N stage. Currently, the prognosis and treatment choices of NPC patients are evaluated primarily with the TNM staging system, which remains a robust prognostic factor.^30^ However, there are significant differences in the prognosis of NPC patients with the same TNM stage, probably because the TNM system does not reflect the survival of patients completely.^31^, ^32^

In addition to anatomical factors, indicators associated with cancer prognoses such as EBV‐DNA,^33^ age,^34^ and gender^34^ were reported to be independently correlated with survival outcomes. Pre‐treatment Epstein–Barr virus DNA level has been found to be predictive for post‐therapy distant failure,^35^ and patients with detectable baseline EBV DNA benefit from induction chemotherapy.^36^ Guo et al. reported that plasma EBV DNA combined with the TNM staging system further improved the prognostic efficacy of NPC patients.^22^ ACE‐27 was able to identify patients at a higher risk of major toxicity and assist in deciding whether to undergo treatment.^37^, ^38^, ^39^ Sommat et al. reported that an ACE27 score of 2 or 3 was significantly associated with overall survival (HR: 2.12, 95% CI: 1.09–4.12) and disease‐free survival (HR: 1.87, 95% CI: 1.02–3.41) in elderly NPC patients, compared with a score of 0 or 1.^40^ Xu et al. reported that female NPC patients had better OS benefits than males when treated with IC,^41^ which is consistent with a previous study that women have a better NPC prognosis compared with men.^42^ Lu Xing et al.^34^ reviewed 2063 consecutive NPC patients and reported that the ratio of the locoregionally advanced stage in female NPC patients (50.6%) was much lower than that of male NPC patients (71.9%), which may lead to better survival outcomes than male patients. In this study, however, the ratio of the locoregionally advanced stage in female and male NPC patients is comparable (85.2% and 88.7%, respectively), which is likely due to clinical decisions that the majority of patients receiving IC are locoregionally advanced stage patients. Previous studies have reported that cigarette smoking is an indicator of prognosis, while the risk of mortality, distant metastasis, locoregional recurrence, and disease progression is significantly higher for former and current smokers than never smokers.^43^ Poor nutritional status is related to the worse overall survival in patients receiving chemotherapy.^44^ As an indicator reflecting the nutritional status of patients, ALB is thought to be related to the prognosis of NPC patients.^45^ Based on multiple sera and clinical prognostic indicators, satisfactory C‐indexes for DSS and OS and good consistency with the calibration plot, this nomogram is suitable for screening elderly NPC patients, which benefits from IC.

One possible explanation for the lack of benefit in medium and low risk group might be that the proportion of patients with T3, T4, N2, N3 was higher in the high risk group (Table 3) than that in the intermediate and low risk group, meaning that there were greater tumor burden or more extensive nodal disease in the high risk group, which has an elevated risk for distant metastasis.^36^ Figure S1 shows that the 5‐year DMFS rate for patients receiving IC and CCRT alone in the high risk group was 80.4% and 71.4%, respectively, *p* = 0.15, and the 5‐year LRRFS rate was 90.6% and 79.2%, respectively, *p* = 0.074. Although not statistically significant, patients receiving IC had better DMFS and LRRFS rate than that in the CCRT alone group, while the survival rate was similar for both treatments in the intermediate‐and low risk group. And this might be able to lead to an overall survival benefit in the high risk group. Besides, given the relatively low mortality rate of NPC patients, the current follow‐up time may not be sufficient to distinguish between differences in treatment outcomes. In addition, since the dose of concurrent cisplatin was not available in our study, the lower relative dose intensify (RDI) of concurrent cisplatin may diminish the effect of CCRT, thus counteracting any benefits from IC. Tan^46^ conducted a clinical trial and randomized 86 NPC patients into the induction GCP (gemcitabine, carboplatin and paclitaxel) plus CCRT group, and 86 NPC patients into the CCRT group. There was no significant difference in the 3‐year OS (94.3% vs. 92.3%, *p* = 0.494) and DMFS (*p* = 0.362) between these two groups, and the relative dose intensity (RDI) for concurrent cisplatin was significantly lower in the induction GCP + CCRT group, compared with the CCRT alone group. Different induction chemotherapy regimens may lead to significantly different survival outcomes. Therefore, there is uncertainty as to which treatment option is appropriate in low and medium risk groups, and clinical trials in these two subgroups are needed in the future.

The main strengths of this study are the establishment of prognostic nomograms based on large‐scale clinical information from real‐world case records and the screening of elderly NPC patients who could benefit from IC by risk stratification. PSM analysis explores the confounding factors and selection bias of observational row data in retrospective studies. Detailed medical records document the process of treatment and the physical status of patients. However, there are some limitations that need to be noted. First, although calibration curves show good agreement and C‐index is satisfactory, external validation is not performed due to the lack of patient data from other hospitals in the endemic region. Second, the harmonization of EBV DNA assay needs to be taken into account since the sensitivity of PCR‐based EBV DNA testing ranges from 53% to 96%.^47^ Third, treatment‐related toxicity is not available by failing to collect information on acute and late toxicity.

## 5 CONCLUSION

Nomogram‐generated hazard stratification based on pre‐treatment plasma EBV DNA, TNM stage, chemistry parameters is strongly predictive of OS and DSS among elderly NPC patients. Compared with CCRT, the addition of IC leads to significantly improved OS and DSS in the high risk group in patients younger than 70 years of ages, indicating that this group may be a clear target population to receive IC + CCRT in clinical practice. IC regimens of TPF and IC cycles of three were recommended. Further investigations are needed for elderly patients in low and medium risk groups.

## ETHICS APPROVAL STATEMENT

This retrospective research was approved by our institution (IRB‐approved number, SL‐B2021‐474‐02).

## PATIENT CONSENT STATEMENT

Clinical information was anonymous, and the requirement of informed consent was waived.

## AUTHOR CONTRIBUTIONS

**Shuiqing He:** Writing – original draft (lead); writing – review and editing (lead). **Yan‐Ling Wu:** Formal analysis (equal); resources (equal). **Yongxiang Gao:** Software (lead). **Danjie He:** Investigation (equal); methodology (equal). **Ying Huang:** Conceptualization (lead); project administration (lead); resources (lead); supervision (lead); validation (lead).

## FUNDING INFORMATION

None.

## CONFLICT OF INTEREST STATEMENT

There are no known conflicts of interest.

## Supporting information


Figure S1:

Table S1:

Table S2:

Supplementary S1

Data S1:

## Data Availability

Cancer Medicine requires, as a condition for publication, that the data supporting the results in the paper will be peer reviewed and archived in an appropriate public repository. Authors are required to provide a data availability statement, including a link to the repository they have used, and to cite the data they have shared. Whenever possible the scripts and other artefacts used to generate the analyses presented in the paper should also be publicly archived. Exceptions may be granted at the discretion of the editor. If sharing data compromises ethical standards or legal requirements then authors are not required to share it.Peer review of empirical data will be conducted to confirm the quality of the shared data, for example, that sample sizes match, that the variables described in the article are present as fields in the data repository, that data is complete; that data is properly labelled and described; and that it has the appropriate metadata for the kind of data being shared.
