# Supplementary figures and images for: Risk stratification and beneficiary selection among elderly nasopharyngeal carcinoma patients from concurrent chemoradiotherapy combined with induction chemotherapy
Source: Cancer Med. 2023 Apr 16;12(9):10536–52. doi: 10.1002/cam4.5789 (PMC10225195; doi:10.1002/cam4.5789)

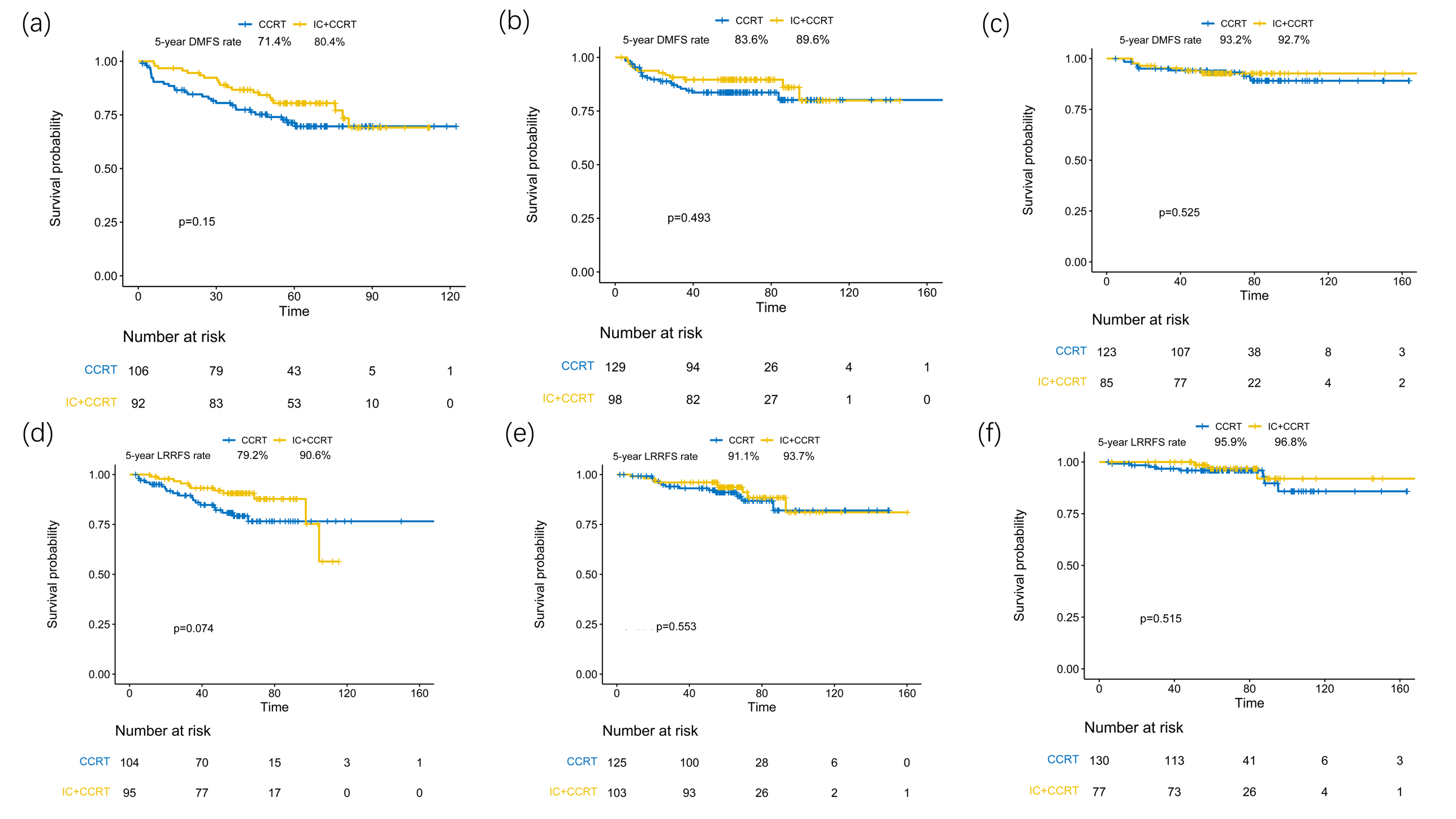

Supplement: Supplementary file 1 — Figure S1: [file CAM4-12-10536-s001.TIF]
