# Supplementary material for: Risk stratification and beneficiary selection among elderly nasopharyngeal carcinoma patients from concurrent chemoradiotherapy combined with induction chemotherapy
Source: Cancer Med. 2023 Apr 16;12(9):10536–52. doi: 10.1002/cam4.5789 (PMC10225195; doi:10.1002/cam4.5789)
Supplement: Supplementary file 2 — Table S1: [file CAM4-12-10536-s002.docx]

Table S1 The baseline characteristics of high, intermediate, and low-risk groups for OS in patients treated with IC plus CCRT or CCRT alone before propensity score matching

| Characteristics | High-risk group | Intermediate-risk group | Low-risk group | P value |
| --- | --- | --- | --- | --- |
|  | n=297 | n=309 | n=303 |  |
|  | No. %^a^ | No. %^a^ | N0. %^a^ |  |
| Gender |  |  |  | <0.001 |
| Male | 247(83.2%) | 248(80.3%) | 198(65.3%) |  |
| Female | 50(16.8%) | 61(28.2%) | 105(34.7%) |  |
| Age |  |  |  | <0.001 |
| 60-64 | 108(36.4%) | 217(70.2%) | 265(87.5%) |  |
| 65-69 | 117(39.4%) | 79(25.6%） | 35(11.6%) |  |
| >=70 | 72(24.2%) | 13(4.2%) | 3(1.0%) |  |
| WHO |  |  |  | 0.106 |
| 3 | 290(97.6%) | 298（96.4%） | 300（99.0%） |  |
| 1&2 | 7(2.4%) | 11（3.6%） | 3(1.0%） |  |
| Smoking |  |  |  | <0.001 |
| no | 126(42.4%) | 158(51.1%) | 235(77.6%) |  |
| yes | 171(57.6%) | 151(48.9%) | 68(22.4%) |  |
| Drinking |  |  |  | <0.001 |
| no | 230(77.4%) | 240(77.7%) | 274(90.4%) |  |
| yes | 67(22.6) | 69(22.3%) | 29(9.6%) |  |
| ACE |  |  |  | <0.001 |
| 0 | 149(50.2%) | 160(51.8%) | 154(50.8%) |  |
| 1 | 111(37.4%) | 140(45.3%) | 149(49.2%) |  |
| 2 | 37(12.5%) | 9(2.9%) |  |  |
| Family history |  |  |  | 0.924 |
| no | 228(76.8%) | 233(75.4%) | 230(75.9%) |  |
| yes | 69(23.2%) | 76(24.6%) | 73(24.1%) |  |
| T stage |  |  |  | <0.001 |
| T1-2 | 22(7.4%) | 54(17.5%) | 103(34.0%) |  |
| T3 | 117(39.4%) | 168(54.4%) | 178(58.7%) |  |
| T4 | 158(53.2%) | 87(28.2%) | 22(7.3%) |  |
| N stage |  |  |  | <0.001 |
| N0-1 | 143(48.1%) | 165(53.4%) | 233(76.9%) |  |
| N2 | 82(27.6%) | 104(33.7%) | 58(19.1%) |  |
| N3 | 72(24.2%) | 40(12.9%) | 12(4.0%) |  |
| Stage |  |  |  | <0.001 |
| II | 6(2.0%) | 31(10.0%) | 73(24.1%) |  |
| III | 82(27.6%) | 158(51.1%) | 197(65.0%) |  |
| IV | 209(70.4%) | 120(38.8%) | 33(10.9%) |  |
| EBV-DNA (copy/ml) ^b^ |  |  |  | <0.001 |
| <=2000 | 68(22.9%) | 132(42.7%） | 246(81.2%) |  |
| >2000 | 229(77.1%) | 177(57.3%) | 57(18.8%) |  |
| HGB (g/L) ^b^ |  |  |  | 0.291 |
| abnormal | 278(93.6%) | 283(91.6%) | 287(94.7%) |  |
| normal | 19(6.4%) | 26(8.4%) | 16(5.3%) |  |
| ALB(g/L) ^b^ |  |  |  | <0.001 |
| 35-55 | 210(70.7%) | 279(90.3%) | 289(95.4%) |  |
| <35 | 87(29.3%) | 30(9.7%) | 14(4.6%) |  |
| LDH ^b^ |  |  |  | <0.001 |
| abnormal | 34(11.4%) | 28(9.1%) | 9(3.0%) |  |
| normal | 263(88.6%) | 281(90.9%) | 294(97.0%) |  |
| CRP(g/L) ^b^ |  |  |  | <0.001 |
| 0-8.2 | 182(61.3%) | 231(74.8%) | 242(79.9%) |  |
| >8.2 | 115(38.7%) | 78(25.2%) | 61(20.1%) |  |
| Treatment |  |  |  | <0.001 |
| IC plus CCRT | 156(52.5%) | 141(45.6%) | 90(29.7%) |  |
| CCRT | 141(47.5%) | 168(54.4%) | 213(70.3%) |  |

^a^: Percentages may not add up to 100% due to rounding

^b^: All variables were measured before treatment

ACE, adult comorbidity evaluation; EBV, Epstein–Barr virus; ALB: albumin; HR, hazard ratio; CRP, C-reactive protein; LDH, lactate dehydrogenase; NPC, nasopharyngeal carcinoma; OS, overall survival; DSS, disease-special survival; WHO, World Health Organization
