# Supplementary material for: Risk stratification and beneficiary selection among elderly nasopharyngeal carcinoma patients from concurrent chemoradiotherapy combined with induction chemotherapy
Source: Cancer Med. 2023 Apr 16;12(9):10536–52. doi: 10.1002/cam4.5789 (PMC10225195; doi:10.1002/cam4.5789)
Supplement: Supplementary file 3 — Table S2: [file CAM4-12-10536-s003.docx]

Table S2 The baseline characteristics of the patients treated with IC plus CCRT or CCRT alone in each risk group for OS after PSM analysis.

| Characteristic | High-risk group | | | Intermediate-risk group | | | Low-risk group | | |
| --- | --- | --- | --- | --- | --- | --- | --- | --- | --- |
|  | CCRT  N = 106 | IC+CCRTN = 86 | p-value^1^ | CCRT  N = 135 | IC+CCRT  N = 114 | p-value^1^ | CCRT  N = 132 | IC+CCRTN = 71 | p-value^1^ |
| Age |  |  | 0.15 |  |  | 0.11 |  |  | 0.7 |
| 60-64 | 31 (29%) | 27 (31%) |  | 90 (67%) | 89 (78%) |  | 126 (95%) | 67 (94%) |  |
| 65-69 | 44 (42%) | 44 (51%) |  | 43 (32%) | 24 (21%) |  | 6 (4.5%) | 4 (5.6%) |  |
| >=70 | 31 (29%) | 15 (17%) |  | 2 (1.5%) | 1 (0.9%) |  | 0 (0%) | 0 (0%) |  |
| Smoking |  |  | 0.4 |  |  | 0.9 |  |  | 0.5 |
| no | 52 (49%) | 37 (43%) |  | 65 (48%) | 56 (49%) |  | 101 (77%) | 57 (80%) |  |
| yes | 54 (51%) | 49 (57%) |  | 70 (52%) | 58 (51%) |  | 31 (23%) | 14 (20%) |  |
| ACE |  |  | 0.3 |  |  | 0.8 |  |  | 0.7 |
| 0 | 52 (49%) | 41 (48%) |  | 68 (50%) | 60 (53%) |  | 67 (51%) | 33 (46%) |  |
| 1 | 43 (41%) | 30 (35%) |  | 62 (46%) | 51 (45%) |  | 65 (49%) | 38 (54%) |  |
| 2 | 11 (10%) | 15 (17%) |  | 5 (3.7%) | 3 (2.6%) |  | 0 (0%) | 0 (0%) |  |
| yes | 21 (20%) | 18 (21%) |  | 33 (24%) | 24 (21%) |  | 34 (26%) | 15 (21%) |  |
| T stage |  |  | 0.7 |  |  | 0.5 |  |  | >0.9 |
| T1-2 | 6 (5.7%) | 4 (4.7%) |  | 25 (19%) | 20 (18%) |  | 45 (34%) | 23 (32%) |  |
| T3 | 44 (42%) | 31 (36%) |  | 73 (54%) | 55 (48%) |  | 82 (62%) | 45 (63%) |  |
| T4 | 56 (53%) | 51 (59%) |  | 37 (27%) | 39 (34%) |  | 5 (3.8%) | 3 (4.2%) |  |
| N stage |  |  | 0.4 |  |  | 0.5 |  |  | 0.3 |
| N0-1 | 51 (48%) | 37 (43%) |  | 76 (56%) | 59 (52%) |  | 103 (78%) | 50 (70%) |  |
| N2 | 28 (26%) | 30 (35%) |  | 44 (33%) | 37 (32%) |  | 25 (19%) | 16 (23%) |  |
| N3 | 27 (25%) | 19 (22%) |  | 15 (11%) | 18 (16%) |  | 4 (3.0%) | 5 (7.0%) |  |
| EBV-DNA(copy/ml) |  |  | 0.8 |  |  | 0.4 |  |  | 0.4 |
| <=2000 | 24 (23%) | 18 (21%) |  | 57 (42%) | 42 (37%) |  | 107 (81%) | 54 (76%) |  |
| >2000 | 82 (77%) | 68 (79%) |  | 78 (58%) | 72 (63%) |  | 25 (19%) | 17 (24%) |  |
| ALB(g/L) |  |  | 0.5 |  |  | >0.9 |  |  | 0.5 |
| 35-55 | 75 (71%) | 65 (76%) |  | 125 (93%) | 106 (93%) |  | 123 (93%) | 68 (96%) |  |
| <35 | 31 (29%) | 21 (24%) |  | 10 (7.4%) | 8 (7.0%) |  | 9 (6.8%) | 3 (4.2%) |  |
| ^1^Pearson's Chi-squared test; Fisher's exact test  ACE, adult comorbidity evaluation; EBV, Epstein–Barr virus; ALB, albumin | | | | | | | | | |
