# Supplementary material for: Risk stratification and beneficiary selection among elderly nasopharyngeal carcinoma patients from concurrent chemoradiotherapy combined with induction chemotherapy
Source: Cancer Med. 2023 Apr 16;12(9):10536–52. doi: 10.1002/cam4.5789 (PMC10225195; doi:10.1002/cam4.5789)
Supplement: Supplementary file 4 — Supplementary S1 [file CAM4-12-10536-s004.docx]

**Supplementary material**

Treatment strategies for nasopharyngeal carcinoma in our hospital

Nasopharyngeal carcinoma (NPC) patients in out hospital was treated using a stratified multi-therapeutic protocol based on the 8th edition of the AJCC/UICC staging system, with intensity-modulated radiotherapy (IMRT) using during the entire course.

Stage I NPC patients received IMRT alone. Patients with stage II received IMRT, with or without concurrent chemotherapy. CCRT or induction chemotherapy (IC) plus CCRT were administered to patients with locoregionally advanced NPC (stage III–IVA; T1, N1-3; T2-4, any N). Since CCRT plus adjuvant chemotherapy has been reported to be related with severe toxicities and low compliance, as well as inferior survival, compared to CCRT alone, patients treated by this method were excluded from this study^1^.

Individualized delineation protocol was used to delineated target volumes slice-by-slice on treatment planning CT scans, which was in accordance with the International Commission on Radiation Units and Measurements reports 50 and 62. The prescribed doses distribution to planning target volume (PTV) of the primary gross tumor volume (GTVnx) was 66-72Gy/28-33F, to the PTV of the GTV of the involved lymph nodes (GTVnd) was 64–70 Gy/28–33F, to the PTV of the high-risk clinical target volume (CTV1) was 60–63 Gy/28–33F, to the PTV of the low-risk clinical target volume (CTV2) was 54–56 Gy/28–33F. Simultaneous integrated boost technique was used to treat all targets. The extension of 5–10 mm beyond the margin of the GTVnx was applied in CTV1 for potential microscopic spread, including the entire nasopharyngeal mucosa and 5 mm into the submucosal region. CTV2 extended 5–10 mm beyond the margin of the CTV1, potentially involving regions and lymphatic regions, unless the CTV2 was adjacent to critical organs, e.g., brain stem and spinal cord, in which case the extension distance was reduced to 3–5 mm.

IC regimens consisted of platinum-based double or triple agents, namely PF, cisplatin–5-fluorouracil (80 mg/m^2^ and 4,000 mg/m^2^, respectively); TP, docetaxel–cisplatin (75 mg/m^2^ and 75 mg/m^2^, respectively); TPF, docetaxel–cisplatin–5-fluorouracil (60 mg/m^2^, 60 mg/m^2^, and 3,000 mg/m^2^, respectively), administrated every three weeks for 2–3 cycles. All chemotherapeutic drugs except for 5-fluorouracil were administered on day 1 of each 21-day cycle, while 5-fluorouracil was given via continuous intravenous infusion on days 1-5; and GP, gemcitabine 1000mg mg/m^2^ on day 1 and 8, and cisplatin 80 mg/m^2^ on day 1, administrated every 3 weeks for three cycles. Concurrent chemotherapy was weekly cisplatin (30–40 mg/m^2^), and cisplatin on weeks 1, 4, and 7 (80–100 mg/m^2^) of IMRT. Moreover, for relapsed or metastatic disease, palliative chemotherapy, intracavitary brachytherapy or salvage surgery were provided if possible.

Reference

1CHEN, L., HU, C. S., CHEN, X. Z., HU, G. Q., CHENG, Z. B., SUN, Y., LI, W. X., CHEN, Y. Y., XIE, F. Y., LIANG, S. B., CHEN, Y., XU, T. T., LI, B., LONG, G. X., WANG, S. Y., ZHENG, B. M., GUO, Y., SUN, Y., MAO, Y. P., TANG, L. L., CHEN, Y. M., LIU, M. Z. & MA, J. 2012. Concurrent chemoradiotherapy plus adjuvant chemotherapy versus concurrent chemoradiotherapy alone in patients with locoregionally advanced nasopharyngeal carcinoma: a phase 3 multicentre randomised controlled trial. Lancet Oncol, 132, 163-171.
